# Supplementary material for: A structural vista of phosducin-like PhLP2A-chaperonin TRiC cooperation during the ATP-driven folding cycle
Source: Nat Commun. 2024 Feb 2;15:1007. doi: 10.1038/s41467-024-45242-x (PMC10837153; doi:10.1038/s41467-024-45242-x)
Supplement: Supplementary file 1 — Supplementary Information [file 41467_2024_45242_MOESM1_ESM.pdf]

Supplemental Information for

A structural vista of phosducin-like PhLP2A-chaperonin TRiC cooperation during the ATP-driven folding cycle

Junsun Park, Hyunmin Kim, Daniel Gestaut, Seyeon Lim, Alexander Leitner, Judith Frydman, Soung-Hun Roh

LIST OF SUPPLEMENTAL INFORMATION

Supplementary Fig. 1. Structural analyses on PhLP2A in an open TRiC complex
Supplementary Fig. 2. Analyses on ATP binding pocket in an open TRiC-PhLP2A complex
Supplementary Fig. 3. Alphafold-adopted model building and crosslinking assay of PhLP2A
Supplementary Fig. 4. The direct competition between PFD and PhLP2A
Supplementary Fig. 5. CryoEM structure of PhLP2A in closed TRiC
Supplementary Fig. 6. Analyses on ATP binding pocket in a closed TRiC-PhLP2A complex
Supplementary Fig. 7. Domain-wise characteristics of PhLP2A in relationship with TRiC
Supplementary Fig. 8. CryoEM structure of PhLP2A-actin encapsulated closed TRiC
Supplementary Fig. 9. Evolutionary analysis on phosducin-like protein family

Supplementary Table 1. CryoEM image collection, map reconstruction, and model refinement
Supplementary Table 2. Crosslinking and mass spectrometry data

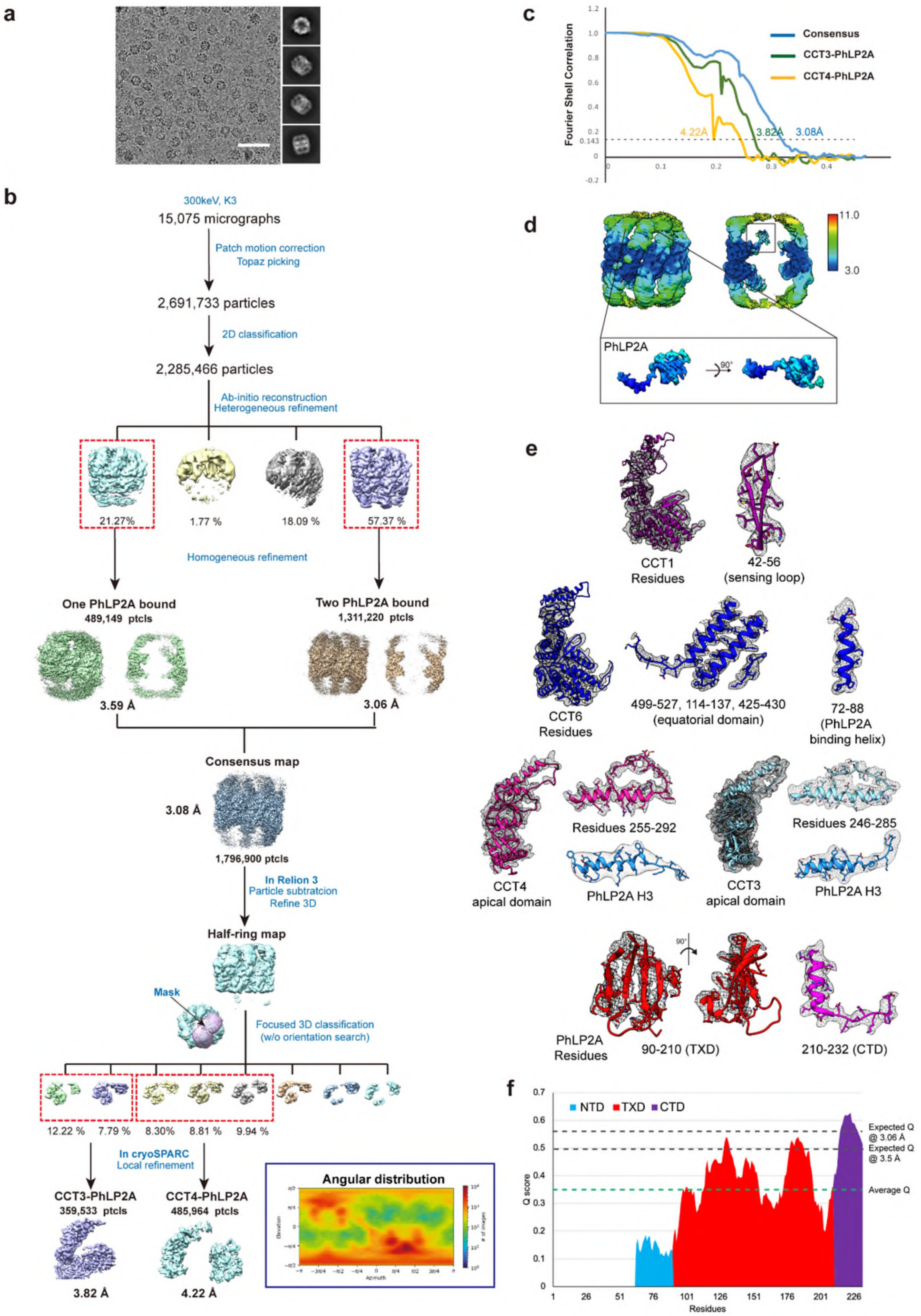

a
b
300keV, K3
15,075 micrographs
Patch motion correction
Topaz picking
2,691,733 particles
2D classification
2,285,466 particles
Ab-initio reconstruction
Heterogeneous refinement
21.27%
1.77 %
18.09 %
57.37 %
Homogeneous refinement
One PhLP2A bound
489,149 ptcls
Two PhLP2A bound
1,311,220 ptcls
3.59 Å
3.06 Å
Consensus map
3.08 Å
1,796,900 ptcls
In Relion 3
Particle subtratcion
Refine 3D
Half-ring map
Mask
Focused 3D classification
(w/o orientation search)
12.22 %
7.79 %
8.30%
8.81 %
9.94 %
In cryoSPARC
Local refinement
CCT3-PhLP2A
359,533 ptcls
CCT4-PhLP2A
485,964 ptcls
3.82 Å
4.22 Å
Angular distribution
c
Fourier Shell Correlation
Consensus
CCT3-PhLP2A
CCT4-PhLP2A
4.22Å
3.82Å
3.08Å
d
11.0
3.0
PhLP2A
90°
e
CCT1
Residues
42-56
(sensing loop)
CCT6
Residues
499-527, 114-137, 425-430
(equatorial domain)
72-88
(PhLP2A
binding helix)
Residues 255-292
CCT4
apical domain
PhLP2A H3
CCT3
apical domain
Residues 246-285
PhLP2A H3
90°
PhLP2A
Residues
90-210 (TXD)
210-232 (CTD)
f
NTD
TXD
CTD
Expected Q
@ 3.06 Å
Expected Q
@ 3.5 Å
Average Q
Q score
Residues

Supplementary Fig. 1. Structural analyses on PhLP2A in an open TRiC complex. **a** Representative electron micrograph of open TRiC-PhLP2A. scale bar, 50 nm. Averaged 2D classes showing top, tilted, and side views are displayed at the right side. **b** Workflow of cryoEM data processing. A diagram for angular distribution of particles for consensus map is displayed. **c** Fourier Shell correlation (FSC) curve of consensus map (blue), local map of CCT3-PhLP2A (green), and local map of CCT4-PhLP2A (yellow) from focused classification. **d** Local resolution map of consensus map and zoom-in view on PhLP2A encapsulated. **e** Map-model fitting of CCT subunits and PhLP2A. CCT1 and its TXD-interacting nucleotide-sensing loop, CCT6 and its equatorial domain and CTD-interacting helix, and apical domain of CCT3, CCT4 are presented with NTD H3, TXD, and CTD of PhLP2A. **f** Q-score graph of PhLP2A in open TRiC. Each domain is color coded as in Fig. 1.

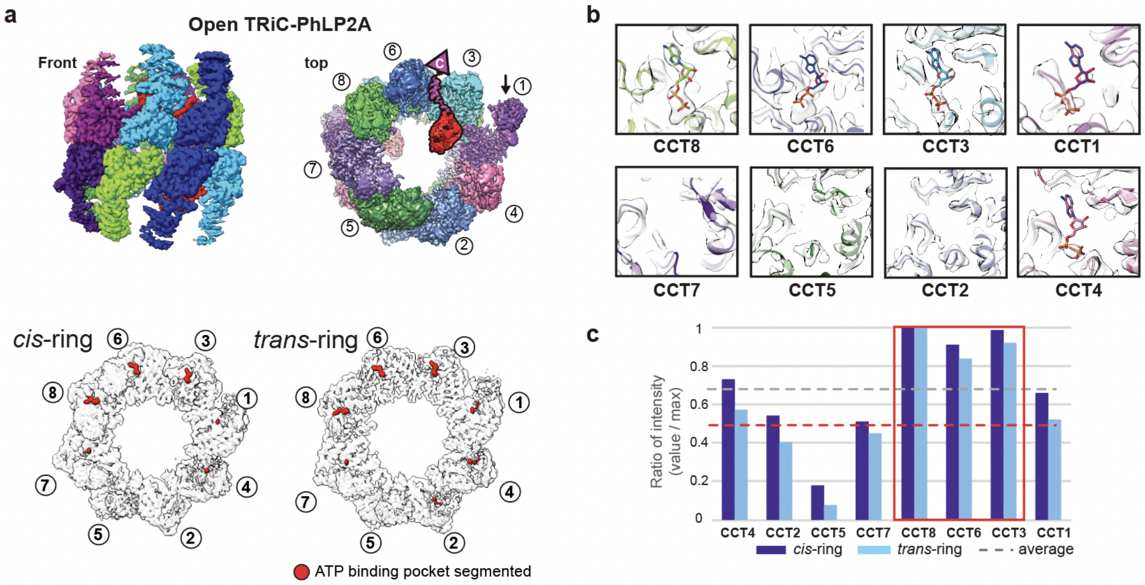

Supplementary Fig. 2 Analyses on ATP binding pocket in an open TRiC-PhLP2A complex a (Top) cryo-EM structure of open TRiC-PhLP2A complex without nucleotide supplemented from this study, (bottom) Density of ATP binding pocket occupied with nucleotide on cis- and trans-ring of TRiC complex. Density corresponds to the nucleotide on each ATP binding pocket is segmented and colored red. **b** Zoom-in view on ATP binding pocket on each CCT subunit, showing different masses depending on different subunits. While CCT8/6/3 show density where nucleotide can fit, other CCT subunits, especially CCT5, show relatively weak densities on the pocket. **c** A graph of the relative intensity of ATP binding pocket on each CCT subunit. The mass of each segmented volume was calculated using ChimeraX Measure volume tool, and the ratio to the maximum volume of the ATP binding pocket was presented. CCT3/6/8 shows significantly higher intensity in comparison with others. Deep blue and sky blue: mass of each CCT subunit in cis-ring and trans-ring, respectively. Light gray dashed line: average of the ratio values, red dashed line: ratio value of 0.5.

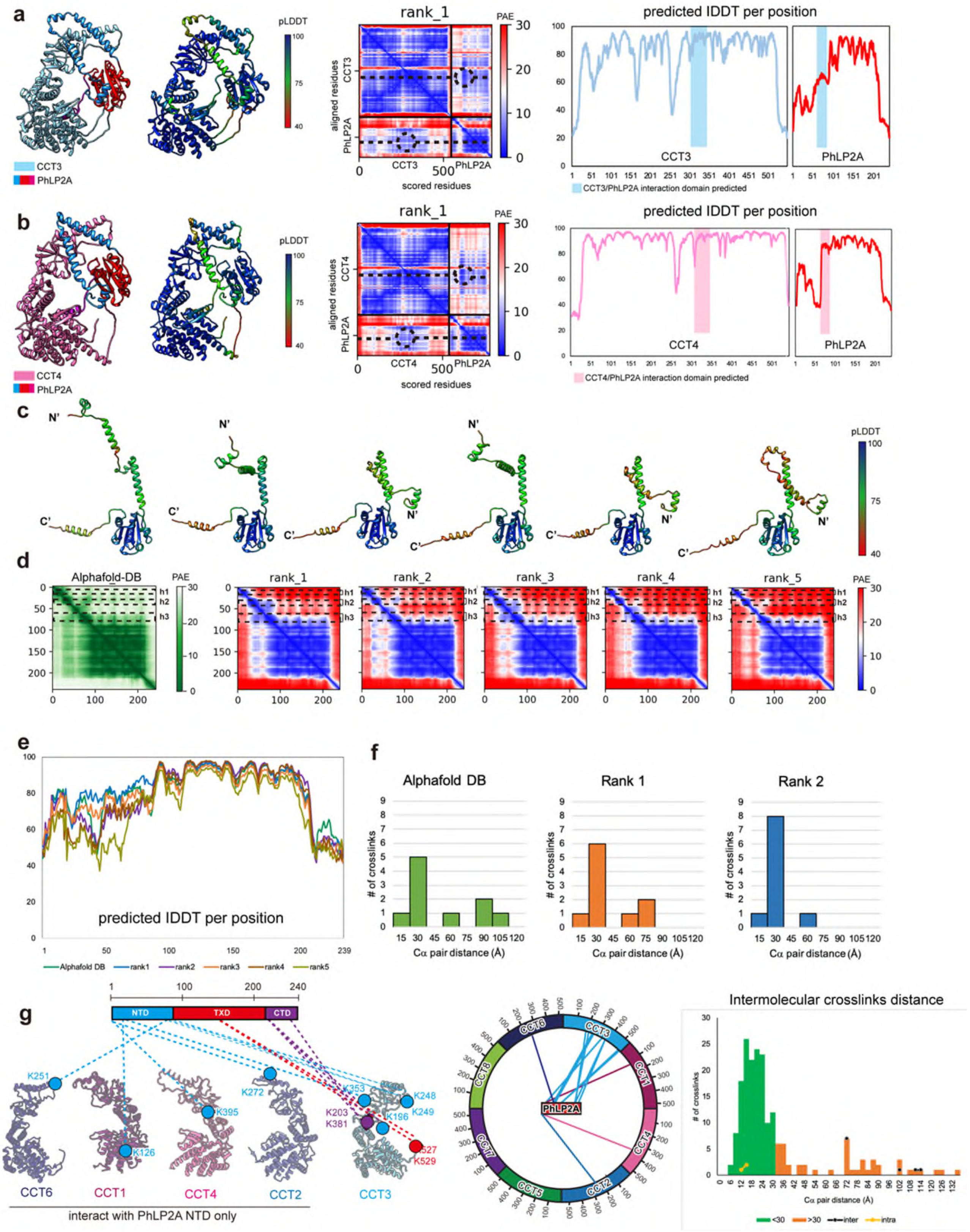
a
CCT3
PhLP2A
pLDDT
rank_1
PAE
aligned residues
scored residues
predicted lDDT per position
CCT3/PhLP2A interaction domain predicted
b
CCT4
PhLP2A
CCT4/PhLP2A interaction domain predicted
c
N'
C'
d
Alphafold-DB
rank_1
rank_2
rank_3
rank_4
rank_5
h1
h2
h3
e
predicted lDDT per position
Alphafold DB
rank1
rank2
rank3
rank4
rank5
f
Alphafold DB
Rank 1
Rank 2
# of crosslinks
Cα pair distance (Å)
g
NTD
TXD
CTD
K251
K126
K395
K272
K353
K248
K249
K196
K203
K381
K527
K529
CCT6
CCT1
CCT4
CCT2
CCT3
interact with PhLP2A NTD only
CCT3
CCT1
CCT4
CCT2
CCT5
CCT7
CCT8
CCT6
PhLP2A
Intermolecular crosslinks distance
# of crosslinks
Cα pair distance (Å)
<30
>30
inter
intra

Supplementary Fig. 3. Alphafold-adopted model building and crosslinking assay of PhLP2A. a Alphafold prediction of a dimeric complex of PhLP2A-CCT3. Models of per-residue confidence score (pLDDT) colored surface, PAE graph with binding surface labeled with a dashed circle, and pLDDT graph are presented. **b** Alphafold-predicted model of PhLP2A-CCT4 and validation graphs. The binding surface between H3 of PhLP2A and H9 on the apical domain of CCT is conserved as observed. **c** Six Alphafold-predicted models of PhLP2A, either Alphafold-DB deposited or predicted models using Colabfold from rank1 to rank 5. Each model is colored with a pLDDT score representing the surface. **d, e** PAE and pLDDT graph of six Alphafold-predicted models. **f** Cα distance graph of intramolecular crosslinks measured on three different Alphafold-predicted models. **g** Schematic diagram of crosslinks between PhLP2A and each CCT subunit. (left) Crosslinks labeled on the model of CCT subunits interacting and schematic diagram of PhLP2A. Each domain of PhLP2A is color-coded as Fig. 1. (middle) Circular diagram of the intermolecular crosslinking result. (right) Cα distance graph of total crosslinks measured on the model of TRiC-PhLP2A complex. Intramolecular crosslinks within PhLP2A and intermolecular crosslinks between PhLP2A and CCTs are indicated as yellow and black dots, respectively.

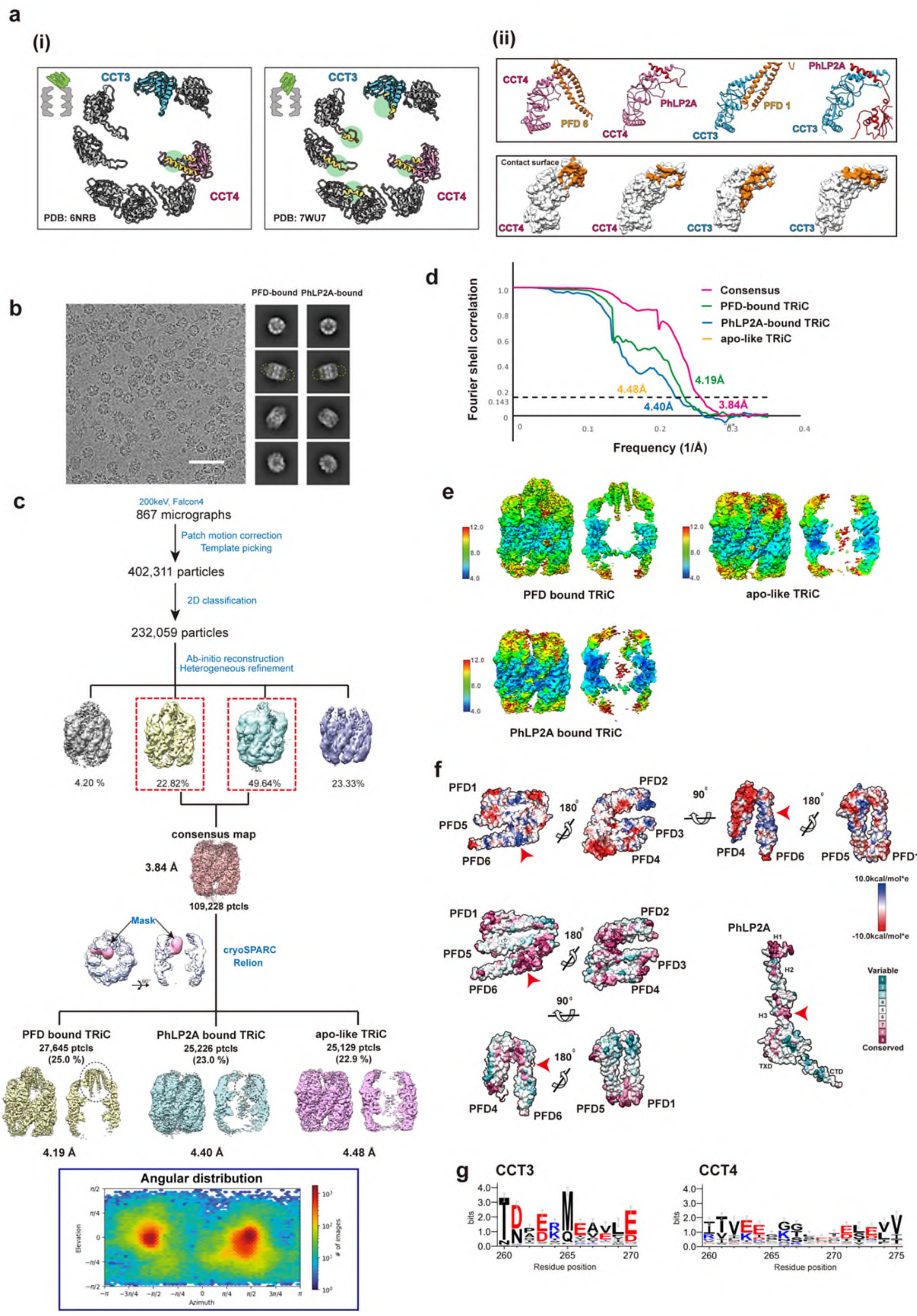
a
(i)
CCT3
CCT4
PDB: 6NRB
PDB: 7WU7
(ii)
CCT4
PFD 6
PhLP2A
PFD 1
CCT3
Contact surface
b
PFD-bound
PhLP2A-bound
c
200keV, Falcon4
867 micrographs
Patch motion correction
Template picking
402,311 particles
2D classification
232,059 particles
Ab-initio reconstruction
Heterogeneous refinement
4.20 %
22.82%
49.64%
23.33%
consensus map
3.84 Å
109,228 ptcls
Mask
cryoSPARC
Relion
PFD bound TRiC
27,645 ptcls
(25.0 %)
PhLP2A bound TRiC
25,226 ptcls
(23.0 %)
apo-like TRiC
25,129 ptcls
(22.9 %)
4.19 Å
4.40 Å
4.48 Å
Angular distribution
Elevation
Azimuth
# of images
d
Fourier shell correlation
Frequency (1/Å)
Consensus
PFD-bound TRiC
PhLP2A-bound TRiC
apo-like TRiC
4.48Å
4.19Å
4.40Å
3.84Å
e
PFD bound TRiC
apo-like TRiC
PhLP2A bound TRiC
f
PFD1
PFD2
PFD3
PFD4
PFD5
PFD6
10.0kcal/mol*e
-10.0kcal/mol*e
PhLP2A
H1
H2
H3
TXD
CTD
Variable
Conserved
g
CCT3
CCT4
bits
Residue position

Supplementary Fig. 4. The direct competition between PFD and PhLP2A. a (i) TRiC contact points for PFD from two distinct conformations (latched, engaged) highlighted with green circles. (ii) The ribbon diagrams of TRiC contact points for PFD subunits (PFD1, 6) and PhLP2A (PhLP2A NTD). The surfaces of CCT3 and CCT4 are shown and the contact area from PFD and PhLP2A is colored in orange. **b** Representative cryoEM micrograph and 2D averages of PFD-bound TRiC and PhLP2A-bound TRiC. scale bar, 50 nm. **c** The workflow of cryoEM data processing (top) and a diagram for particle angular distribution (bottom). **d** The Fourier shell correlation (FSC) curve. **e** Local resolution map shown in color coded. **f** (Top) The surface charge distribution of PFD subunits and (bottom) the surface of PFD and PhLP2A colored by residue conservation score. The red arrow indicates PFD and PhLP2A binding site. Each conservation score of PFD subunits and CCT subunits is independently calculated and normalized using Consurf. **g** Logo plot of the conservation of CCT3 and CCT4 making contacts with PhLP2A or PFD.

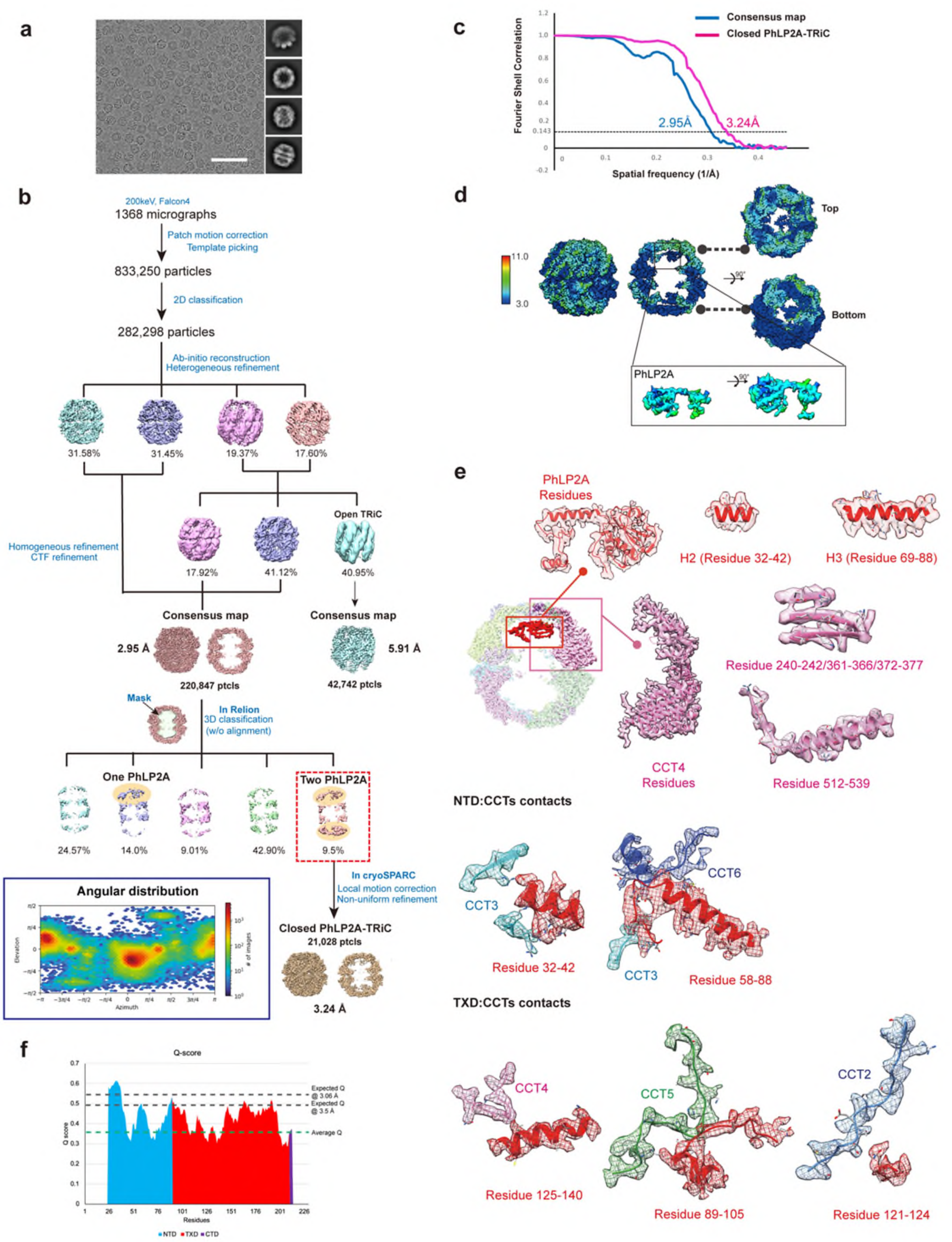

a
b
200keV, Falcon4
1368 micrographs
Patch motion correction
Template picking
833,250 particles
2D classification
282,298 particles
Ab-initio reconstruction
Heterogeneous refinement
31.58%
31.45%
19.37%
17.60%
Homogeneous refinement
CTF refinement
17.92%
41.12%
Open TRiC
40.95%
Consensus map
2.95 Å
220,847 ptcls
Consensus map
5.91 Å
42,742 ptcls
Mask
In Relion
3D classification
(w/o alignment)
One PhLP2A
Two PhLP2A
24.57%
14.0%
9.01%
42.90%
9.5%
In cryoSPARC
Local motion correction
Non-uniform refinement
Closed PhLP2A-TRiC
21,028 ptcls
3.24 Å
Angular distribution
Elevation
Azimuth
# of images
c
Fourier Shell Correlation
Spatial frequency (1/Å)
Consensus map
Closed PhLP2A-TRiC
2.95Å
3.24Å
d
Top
Bottom
PhLP2A
e
PhLP2A
Residues
H2 (Residue 32-42)
H3 (Residue 69-88)
Residue 240-242/361-366/372-377
CCT4
Residues
Residue 512-539
NTD:CCTs contacts
CCT3
Residue 32-42
CCT6
CCT3
Residue 58-88
TXD:CCTs contacts
CCT4
Residue 125-140
CCT5
Residue 89-105
CCT2
Residue 121-124
f
Q-score
Q score
Residues
Expected Q @ 3.06 Å
Expected Q @ 3.5 Å
Average Q
NTD
TXD
CTD

Supplementary Fig. 5. CryoEM structure of PhLP2A in closed TRiC. a Electron micrograph of closed TRiC-PhLP2A. Representative 2D classes of tilted, top, and side view are shown at right. scale bar, 50 nm. **b** Data processing workflow of PhLP2A-closed TRiC and a diagram for particle angular distribution. **c** Fourier Shell Correlation (FSC) curve of structures of PhLP2A-closed TRiC. Consensus map and the map from focused classification are color coded in dodger blue and deep pink, respectively. **d** Local resolution map of PhLP2A-closed TRiC and zoom-in view on PhLP2A encapsulated inside a folding chamber. **e** Map-model fitting. CCT4 and newly resolved H2 and H3 of PhLP2A are shown individually. Map-model fitting on contacts between NTD:CCT and TXD:CCT are presented together. **f** Q-score graph of PhLP2A to corresponding electron density.

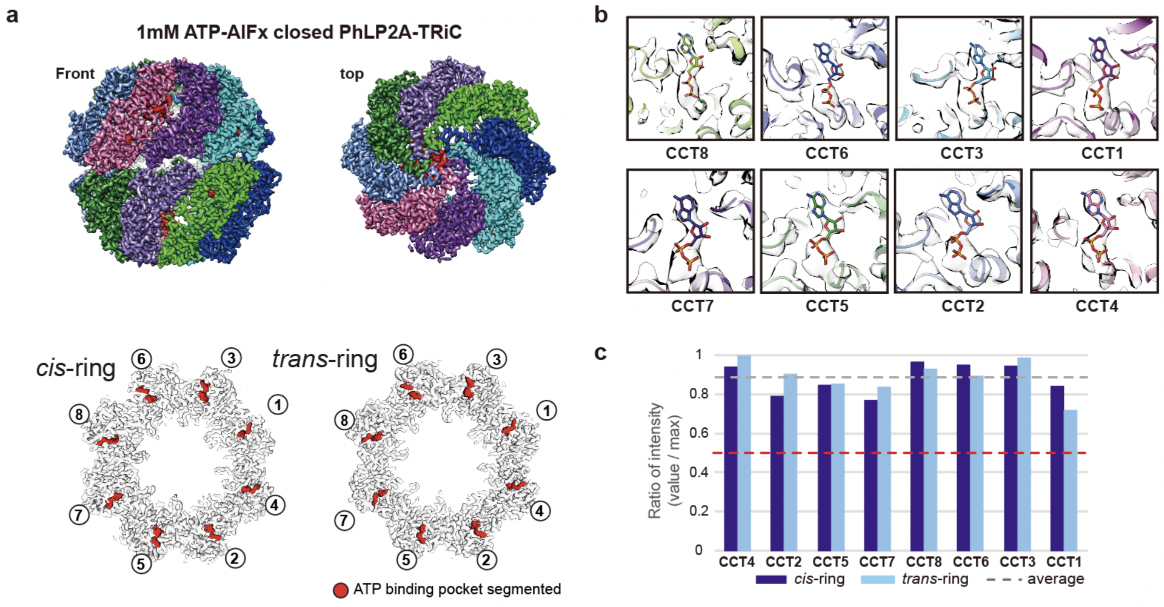

Supplementary Fig. 6. Analyses on ATP binding pocket in a closed TRiC-PhLP2A complex a (Top) cryo-EM structure of closed TRiC-PhLP2A induced by 1mM ATP/AlFx. (bottom) Density of ATP binding pocket occupied with nucleotide on cis- and trans-ring of TRiC complex. **b** Zoom-in view on ATP binding pocket on each CCT subunit. **c** A graph of the relative intensity of ATP binding pocket on each CCT subunit. The mass of each segmented volume and the ratio to the maximum value from the molecule are calculated and presented in Supplementary Fig. 2c.

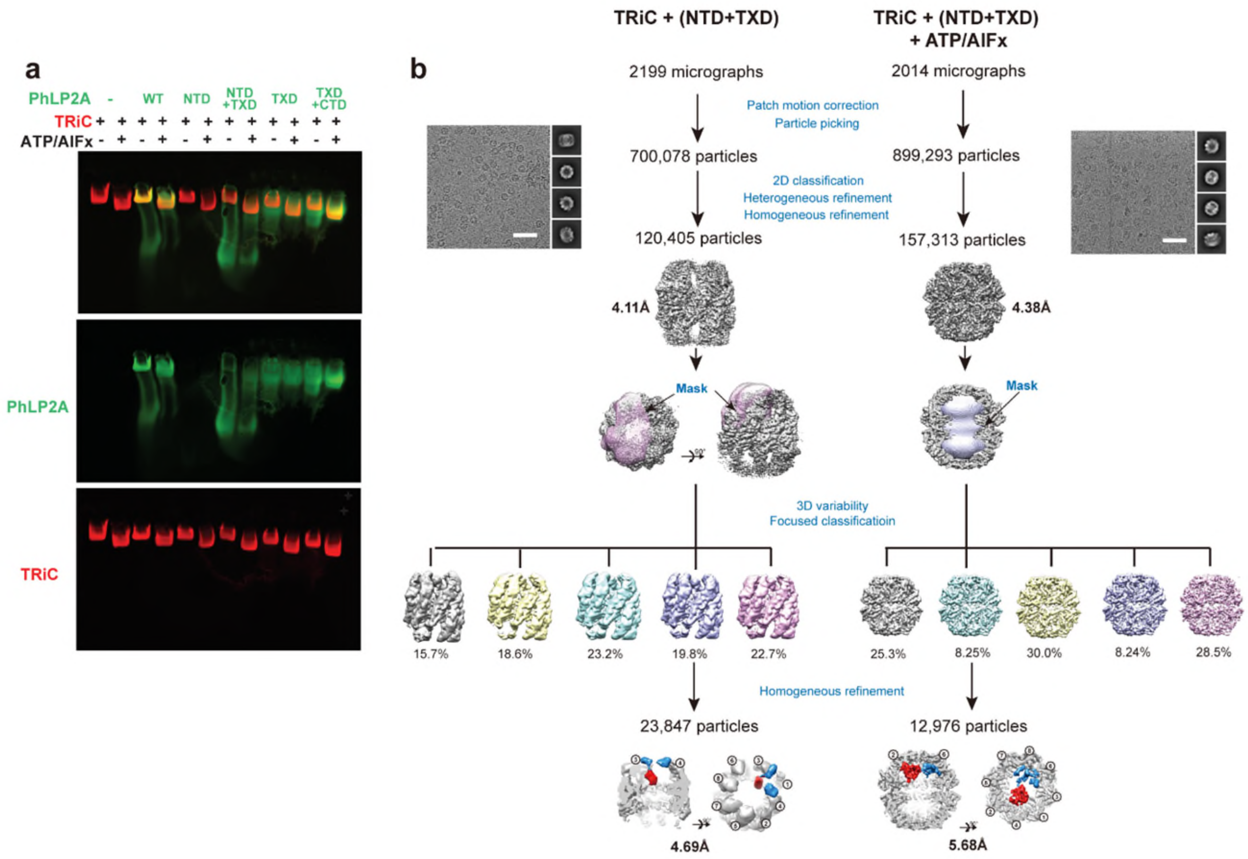

c

No. of particles	Open TRiC					Closed TRiC				
	PhLP2A WT		NTD+TXD	TXD	TXD+CTD	PhLP2A WT		NTD+TXD	TXD	TXD+CTD
	1 bound	2 bound				1 bound	2 bound			
Consensus map (A)	1,796,900		120,405	15,519	27,116	220,847		157,313	24,924	39,112
Final map (B)	485,932	1,310,968	23,847	15,519	10,821	30,911	21,028	12,976	24,924	39,112
Percentage (A/B*100)	27.04%	72.96%	19.8%	-	39.91%	14.00%	9.52%	8.25%	-	-

Supplementary Fig. 7. Domain-wise characteristics of PhLP2A in relationship with TRiC. **a** Native-PAGE based binding assay between TRiC and PhLP2A constructs. **b** CryoEM data-processing workflow of TRiC incubated with NTD-TXD protein in open (left) and closed (right) states with representative micrograph and 2D class averages. scale bar, 50 nm. **c** Table showing population and percentages of encapsulated PhLP2A WT or different constructs of PhLP2A in TRiC at open and closed states.

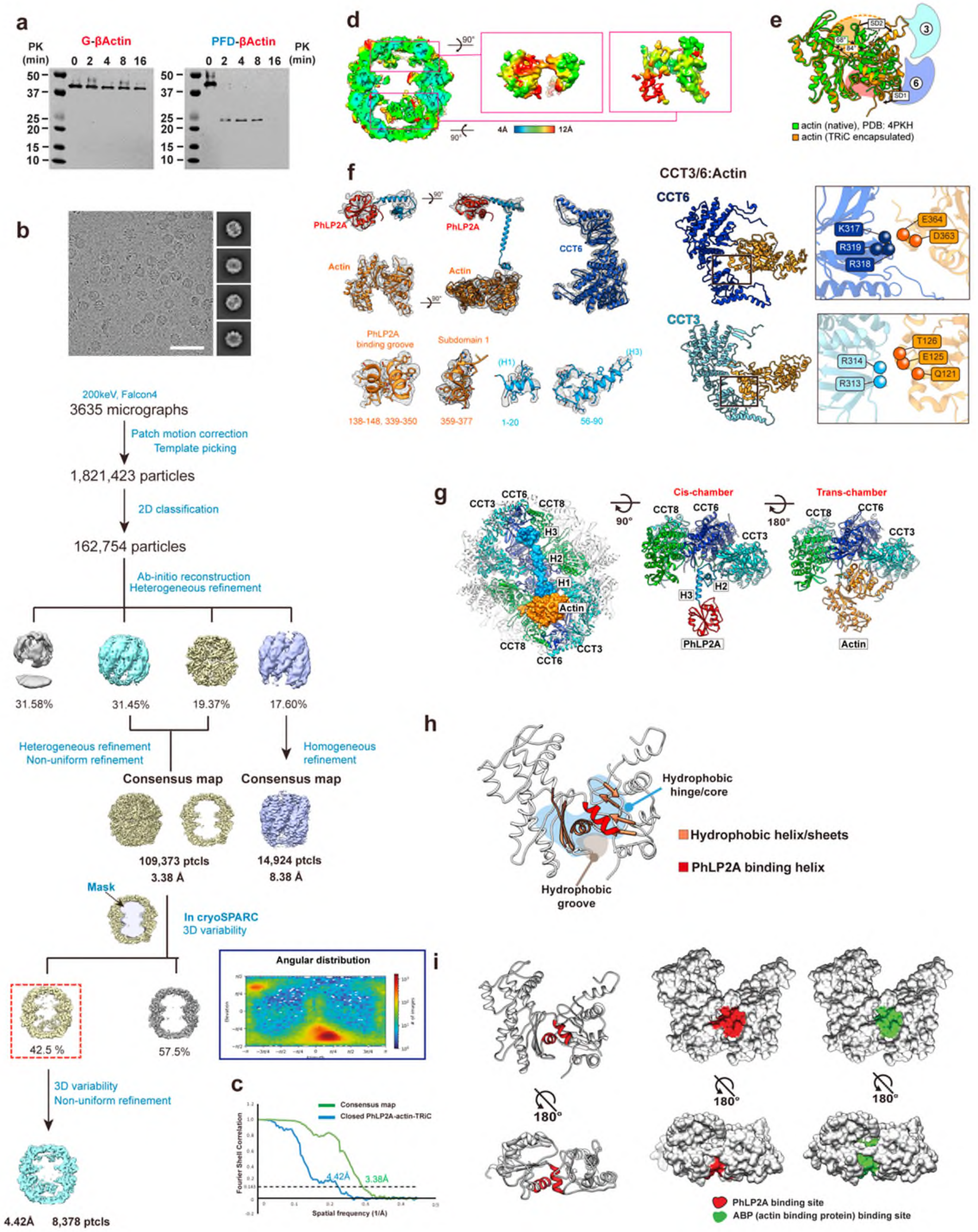
a
G-βActin
PFD-βActin
PK (min)
0 2 4 8 16
50
37
25
20
15
10
b
200keV, Falcon4
3635 micrographs
Patch motion correction
Template picking
1,821,423 particles
2D classification
162,754 particles
Ab-initio reconstruction
Heterogeneous refinement
31.58%
31.45%
19.37%
17.60%
Heterogeneous refinement
Non-uniform refinement
Homogeneous refinement
Consensus map
Consensus map
109,373 ptcls
3.38 Å
14,924 ptcls
8.38 Å
Mask
In cryoSPARC
3D variability
42.5 %
57.5%
3D variability
Non-uniform refinement
4.42Å
8,378 ptcls
Angular distribution
c
Consensus map
Closed PhLP2A-actin-TRiC
Fourier Shell Correlation
Spatial frequency (1/Å)
4.42Å
3.38Å
d
4Å
12Å
e
actin (native), PDB: 4PKH
actin (TRiC encapsulated)
f
PhLP2A
Actin
CCT6
PhLP2A binding groove
Subdomain 1
138-148, 339-350
359-377
1-20
56-90
CCT3/6:Actin
CCT6
CCT3
K317
R319
R318
E364
D363
R314
R313
T126
E125
Q121
g
CCT3
CCT6
CCT8
H3
H2
H1
Actin
Cis-chamber
Trans-chamber
PhLP2A
h
Hydrophobic hinge/core
Hydrophobic helix/sheets
PhLP2A binding helix
Hydrophobic groove
i
180°
PhLP2A binding site
ABP (actin binding protein) binding site

Supplementary Fig. 8. CryoEM structure of PhLP2A-actin encapsulated closed TRiC. a Protein kinase assays on globular actin and PFD-bound actin. While globular actin is protected from proteolysis, PFD-actin is cleaved after incubated with protein kinases. Actin is immunoblotted by anti-actin antibody. **b** (top) Representative cryoEM micrograph and 2D class averages of PhLP2A-actin-TRiC in closed conformation. scale bar, 50 nm and (bottom) Data processing workflow of PhLP2A-actin-TRiC in closed conformation. A diagram of angular distribution for the particles is displayed. **c** FSC curves of consensus map and reconstructed map of PhLP2A-actin-TRiC after focused classification. **d** Local resolution estimation of reconstructed map and zoom-in view on encapsulated PhLP2A (left) or actin (right). **e** Comparison between models of native actin (PDB: 4PKH) and encapsulated actin. **f** Map-model fitting of the reconstructed map. Global fitting of PhLP2A, actin, and CCT6 are presented. Residues of actin interacting with CCT3 or CCT6 are also displayed. **g** The slice view of actin and PhLP2A surface in the TRiC chamber. PhLP2A and actin are shown from the outer chamber view with CCT3/6/8. **h** The interdomain of encapsulated actin. Hydrophobic sheets and helix are colored in orange while the PhLP2A binding helix is colored in red. The hydrophobic hinge/core and hydrophobic groove are represented. **i** The top and side view of PhLP2A binding site and ABP binding site representation on actin surface. Each surface is colored in red and green, respectively.

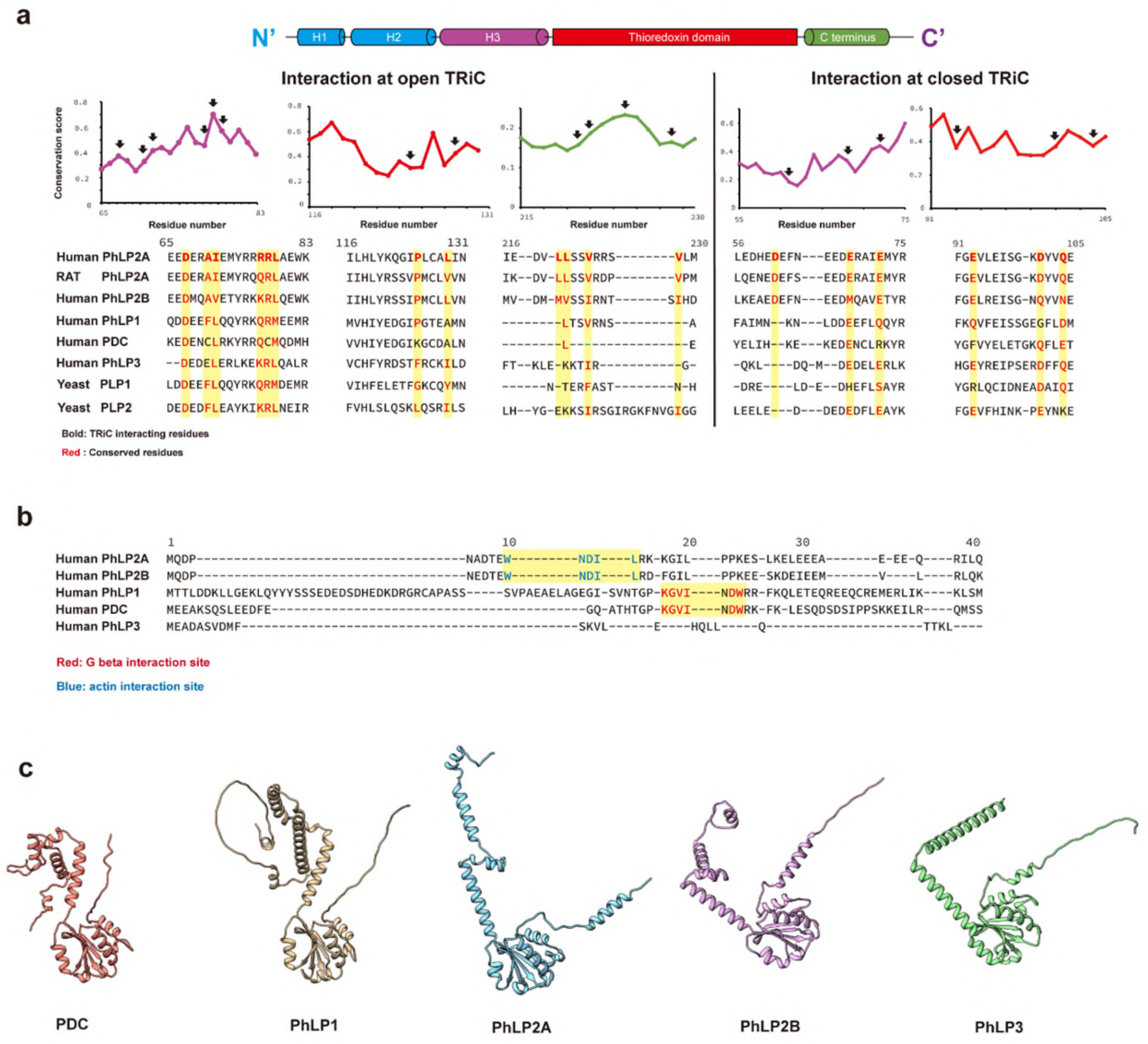

Supplementary Fig. 9. Evolutionary analysis on phosducin-like protein family. a Representative sequence alignments for human PhLP families, rat PhLP2A and yeast PLPs. Plots on the left shows interacting residues in open TRiC state while plots on the right shows interacting regions in closed TRiC state. The average conservation scores of 71 phosducin (PDC) and PhLPs are plotted above. The interacting residues are indicated as black arrows in the plot. Human PhLP2A residues interacting with TRiC are colored in bold red and conserved residues in other phosducin and PhLPs are indicated as red. Left panel shows residues interacting within open TRiC while the right panel shows residues interacting within closed TRiC. **b** Sequence alignment among NTDs of 5 human phosducin families. Red indicates G beta binding site and blue indicates actin binding site. **c** Model of Alphafold-predicted 5 human phosducin and PhLPs from Alphafold DB.

LEGENDS TO SUPPLEMENTAL TABLES

Supplementary Table 1. CryoEM image collection, map reconstruction, and model refinement. List of cryoEM image collection information and map reconstruction, and model refinement statistics.

CryoEM image collection and map processing						
	**TRiC-PhLP2A**	**TRiC-PhLP2A (CCT3 focused)**	**TRiC-PhLP2A (CCT4 focused)**	**TRiC-PhLP2A-ATP/AlFx**	**TRiC-PhLP2A-actin-ATP/AlFx**	**TRiC-PFD TRiC**
**Voltage (kV)**	300			200	200	200
**Total electron exposure ($e^-/Å^2$)**	50			40	40	40
**Defocus range (μm)**	-1.0~-1.8			-1.2~-1.8	-1.2~-1.8	-1.2~-1.8
**Pixel size (Å)**	1.13			1.1	1.1	1.1
**Symmetry imposed**	C1			C1	C1	C1
**Particle images (no.)**	1,796,900	359.533	485,964	21,028	8,378	50,832
**Map resolution (Å) FSC threshold (0.143)**	3.1	3.82	4.22	3.24	4.42	4.19
**EMDB ID**	35284	35199	35280	35122	35335	35284 (An additional map)

Model refinement					
	**TRiC-PhLP2A**	**TRiC-PhLP2A (CCT3 focused)**	**TRiC-PhLP2A (CCT4 focused)**	**TRiC-PhLP2A-ATP/AlFx**	**TRiC-PhLP2A-actin-ATP/AlFx**
**Initial model used** (PDB code)	6NR8	6NR8	6NR8	7NVM, 7TUB	7LUM 7NVM
**Model composition**					
Non-hydrogen atoms	65,141	5,287	5,177	67,629	68,540
Protein residues	8,483	670	675	8,763	8,948
Ligands	ADP:10	ADP:1		MG: 16<br>ADP: 16<br>AF_3: 16	
**R.m.s. deviations**					
Bond lengths (Å)	0.003	0.003	0.005	0.003	0.003
Bond angles (°)	0.872	0.697	0.828	0.627	0.710
**Validation**					
MolProbity score	2.01	1.95	2.36	1.96	2.29
Clashscore	4.20	7.55	18.09	11.10	23.74
Rotamer outliers (%)	1.78	0.17	0.17	0.00	0.01
**Ramachandran plot**					
Favored (%)	87.16	90.39	87.78	94.13	93.64
Allowed (%)	12.10	9.61	12.07	5.83	6.36
Outliers (%)	0.75	0.00	0.17	0.03	0.00
**PDB ID**	8I9U	8I6J	8I9Q	8I1U	8IB8
