# Supplementary material for: A structural vista of phosducin-like PhLP2A-chaperonin TRiC cooperation during the ATP-driven folding cycle
Source: Nat Commun. 2024 Feb 2;15:1007. doi: 10.1038/s41467-024-45242-x (PMC10837153; doi:10.1038/s41467-024-45242-x)
Supplement: Supplementary file 3 — Description of Additional Supplementary Files [file 41467_2024_45242_MOESM3_ESM.pdf]

File name: Supplementary Data 1.

Description: Crosslinking and mass spectrometry data.

File name: Supplementary Movie 1.

Description: Domain-wise molecular contacts of PhLP2A to open TRiC; Related to figure 1 and 2. This movie demonstrates the local density and atomic model of open TRiC and PhLP2A.

File name: Supplementary Movie 2.

Description: Domain-wise molecular contacts of PhLP2A to closed TRiC; Related to figure 4. This movie demonstrates the local density and atomic model of PhLP2A in closed TRiC.

File name: Supplementary Movie 3.

Description: The global relocation of PhLP2A upon TRiC ATP hydrolysis; Related to figure 4. The movie shows the model trajectory from the open to the closed state of TRiC.

File name: Supplementary Movie 4.

Description: Substrate induced conformational changes of PhLP2A NTD and molecular contacts of PhLP2A to TRiC and substrate; Related to figure 6. The movie shows the model trajectory from substrate-free TRiC to actin-TRiC in closed states and a zoom-in view of the molecular contacts of PhLP2A to TRiC and actin substrate.
