# Supplementary material for: A structural vista of phosducin-like PhLP2A-chaperonin TRiC cooperation during the ATP-driven folding cycle
Source: Nat Commun. 2024 Feb 2;15:1007. doi: 10.1038/s41467-024-45242-x (PMC10837153; doi:10.1038/s41467-024-45242-x)
Supplement: Supplementary file 9 — Reporting Summary [file 41467_2024_45242_MOESM9_ESM.pdf]

nature portfolio

Corresponding author(s): Soung-Hun Roh, Judith Frydman

Last updated by author(s): Dec 13, 2023

# Reporting Summary

Nature Portfolio wishes to improve the reproducibility of the work that we publish. This form provides structure for consistency and transparency in reporting. For further information on Nature Portfolio policies, see our Editorial Policies and the Editorial Policy Checklist.

## Statistics

For all statistical analyses, confirm that the following items are present in the figure legend, table legend, main text, or Methods section.

| n/a | Confirmed | |
|---|---|---|
| ☐ | ☒ | The exact sample size (*n*) for each experimental group/condition, given as a discrete number and unit of measurement |
| ☐ | ☒ | A statement on whether measurements were taken from distinct samples or whether the same sample was measured repeatedly |
| ☒ | ☐ | The statistical test(s) used AND whether they are one- or two-sided<br>*Only common tests should be described solely by name; describe more complex techniques in the Methods section.* |
| ☒ | ☐ | A description of all covariates tested |
| ☒ | ☐ | A description of any assumptions or corrections, such as tests of normality and adjustment for multiple comparisons |
| ☐ | ☒ | A full description of the statistical parameters including central tendency (e.g. means) or other basic estimates (e.g. regression coefficient) AND variation (e.g. standard deviation) or associated estimates of uncertainty (e.g. confidence intervals) |
| ☒ | ☐ | For null hypothesis testing, the test statistic (e.g. *F*, *t*, *r*) with confidence intervals, effect sizes, degrees of freedom and *P* value noted<br>*Give P values as exact values whenever suitable.* |
| ☒ | ☐ | For Bayesian analysis, information on the choice of priors and Markov chain Monte Carlo settings |
| ☒ | ☐ | For hierarchical and complex designs, identification of the appropriate level for tests and full reporting of outcomes |
| ☒ | ☐ | Estimates of effect sizes (e.g. Cohen's *d*, Pearson's *r*), indicating how they were calculated |

*Our web collection on statistics for biologists contains articles on many of the points above.*

## Software and code

Policy information about availability of computer code

| | |
|---|---|
| Data collection | We used Thermofisher EPU version 2.10.0.5REL for cryoEM image data collection, and HHMER, NCBI (cd02957) for protein sequence pooling. Thermo Xcalibur, version 4.3 was used for XL-MS data acquisition. |
| Data analysis | We used Relion 4.0, cryoSPARC v3.3 for cryoEM data analysis, Colabfold, Coot 0.9.6 EL, and Phenix 1.19.2 for protein model building, Q-score analysis implemented in Chimera for model validation, UCSF Chimera v 1.15, UCSF Chimera X v 1.3 for visualization, and t-coffee, Weblogo3, Fasttree, and dendroscope for sequence analysis. Consurf for residue conservation score. xQuest, version 2.1.5, was used for XL-MS data analysis. |

For manuscripts utilizing custom algorithms or software that are central to the research but not yet described in published literature, software must be made available to editors and reviewers. We strongly encourage code deposition in a community repository (e.g. GitHub). See the Nature Portfolio guidelines for submitting code & software for further information.

# Data

Policy information about availability of data

All manuscripts must include a data availability statement. This statement should provide the following information, where applicable:

- Accession codes, unique identifiers, or web links for publicly available datasets
- A description of any restrictions on data availability
- For clinical datasets or third party data, please ensure that the statement adheres to our policy

The 3D cryoEM density maps have been deposited in the Electron Microscopy Data Bank under the accession number EMD-35284 (TRiC-PhLP2A open consensus), 35199 (TRiC-PhLP2A open, CCT3 focused), 35280 (TRiC-PhLP2A open, CCT4 focused), 35122 (TRiC-PhLP2A-ATP/AlFx), 35335 (TRiC-PhLP2A-actin-ATP/AlFx). PhLP2A-PFD-TRiC experiments maps (apo-like TRiC, PFD bound TRiC, PhLP2A bound TRiC) are deposited as additional maps in EMD-35284. Truncated PhLP2A-TRiC experiments maps in open or closed states are deposited as additional maps in EMD-35284 and 35122, respectively. Coordinates have been deposited in the Protein Data Bank under the accession number PDB (8I9U, 8I6J, 8I9Q, 8I1U, 8IB8). The mass spectrometry proteomics data have been deposited to the ProteomeXchange Consortium via the PRIDE partner repository with the dataset identifier PXD 040144.

# Research involving human participants, their data, or biological material

Policy information about studies with human participants or human data. See also policy information about sex, gender (identity/presentation), and sexual orientation and race, ethnicity and racism.

| | |
|---|---|
| Reporting on sex and gender | This information has not been collected |
| Reporting on race, ethnicity, or other socially relevant groupings | This information has not been collected |
| Population characteristics | This information has not been collected |
| Recruitment | This information has not been collected |
| Ethics oversight | This information has not been collected |

Note that full information on the approval of the study protocol must also be provided in the manuscript.

# Field-specific reporting

Please select the one below that is the best fit for your research. If you are not sure, read the appropriate sections before making your selection.

☒ Life sciences ☐ Behavioural & social sciences ☐ Ecological, evolutionary & environmental sciences

For a reference copy of the document with all sections, see nature.com/documents/nr-reporting-summary-flat.pdf

# Life sciences study design

All studies must disclose on these points even when the disclosure is negative.

| | |
|---|---|
| Sample size | For each cryoEM structure, cryoEM data analysis are performed with several thousand micrographs (shown in Figure S1-S6). The number of micrographs were determined based on our available microscope time and particle numbers. Particle numbers were roughly determined for the effective classification and observing rare subpopulations based on the previous studies (1 million ~ 2 million). For sequence alignment and analysis, number of sequences were determined based on available NCBI protein family information (cd02957) and HMMER hits. |
| Data exclusions | Micrographs with bad CTF resolution or ice contamination or bad particle distributions were excluded from the processing. For sequence alignment and analysis, too many hits from mammalian systems were excluded in order to avoid biased sequence analysis and to cover overall eukaryotic system. |
| Replication | Each cryoEM maps are reconstructed using millions of particles and processed carefully with different processing pipeline (relion, cryosparc) to check reproducibility and therefore has inherent replication. Each native gel experiments were performed in duplicate or more and confirm the reproducibility successfully. Mass analysis were performed in single. |
| Randomization | Random particle subsets were used during the 3D classification/refinement steps with low pass filtered initial maps to avoid biased classification. Each dataset was randomly split in two halves and refined independently (Gold standard) calculating Fourier Shell Correlation at 0.143. Randomization is not relevant to other experiments as we did not perform the statistical analysis. |
| Blinding | No blinding was necessary since large data (micrographs) acquisition was performed and processing was unbiased following current standard pipeline in cryoEM field. Blinding is not relevant to other experiments as we did not perform the statistical analysis. |

# Reporting for specific materials, systems and methods

We require information from authors about some types of materials, experimental systems and methods used in many studies. Here, indicate whether each material, system or method listed is relevant to your study. If you are not sure if a list item applies to your research, read the appropriate section before selecting a response.

## Materials & experimental systems

| n/a | Involved in the study |
|---|---|
| ☐ | ☒ Antibodies |
| ☐ | ☒ Eukaryotic cell lines |
| ☒ | ☐ Palaeontology and archaeology |
| ☒ | ☐ Animals and other organisms |
| ☒ | ☐ Clinical data |
| ☒ | ☐ Dual use research of concern |
| ☒ | ☐ Plants |

## Methods

| n/a | Involved in the study |
|---|---|
| ☒ | ☐ ChIP-seq |
| ☒ | ☐ Flow cytometry |
| ☒ | ☐ MRI-based neuroimaging |

## Antibodies

Antibodies used

anti-His mouse (MA1-21315 Thermo Fisher Scientific), anti-CCT4 Rabbit Invitrogen (MA5-44927 Thermo Fisher Scientific), anti-Actin (JLA20 – https://dshb.biology.uiowa.edu/JLA20), IRDye 800RD Goat anti-rabbit IgG (Cat# 926-32211, RRID: AB_621843), IRDye 680RD Donkey anti-rabbit IgG (Cat# 926-68073, RRID: AB_10954442), IRDye 800RD Donkey anti-mouse IgG (Cat# 926-32212, RRID: AB_621847)

Validation

Antibodies were validated by the manufacturers as follows;
- anti-His mouse: Immunofluorescent analysis of 6x-His Tag was performed using 70% confluent HEK-293 cells transfected with V5-H3-His construct.
- anti-CCT4 Rabbit: Western blot analysis of TCP1 delta in different lysates. Exposure time: 58 seconds; 4-20% SDS-PAGE gel. Proteins were transferred to a PVDF membrane and blocked with 5% NFDM/TBST for 1 hour at room temperature. Samples were incubated in TCP1 delta Monoclonal antibody (Product # MA5-44927) using a dilution of 1:1,000 in 5% NFDM/TBST at room temperature for 2 hours followed by Goat Anti-Mouse IgG - HRP secondary antibody at a dilution of 1:100,000 for 1 hour at room temperature.
- anti-Actin: This antibody recognizes all isoforms of actin over a broad range of species. JLA20 was deposited to the DSHB by Lin, J.J.-C. (DSHB Hybridoma Product JLA20)
- IRDye 800RD Goat anti-rabbit IgG: This antibody was tested by dot blot and and/or solid-phase adsorbed for minimal cross-reactivity with human, mouse, rat, sheep, and chicken serum proteins, but may cross-react with immunoglobulins from other species. The conjugate has been specifically tested and qualified for Western blot and In-Cell Western™ Assay applications.
- IRDye 680RD Donkey anti-rabbit IgG: This antibody was tested by ELISA and/or solid-phase adsorbed to ensure minimal cross-reactivity with bovine, chicken, goat, guinea pig, hamster, horse, human, mouse, rat, and sheep serum proteins, but may cross-react with immunoglobulins from other species. The conjugate has been specifically tested and qualified for Western blot and In-Cell Western™ Assay applications.
- IRDye 800RD Donkey anti-mouse IgG: This antibody was tested by ELISA and/or solid-phase adsorbed to ensure minimal cross-reactivity with bovine, chicken, goat, guinea pig, horse, human, rabbit, and sheep serum proteins, but may cross-react with immunoglobulins from other species. The conjugate has been specifically tested and qualified for Western blot and In-Cell Western™ Assay applications.

## Eukaryotic cell lines

Policy information about cell lines and Sex and Gender in Research

Cell line source(s)

SF9 cells and High Five cells (Thermo Fisher Scientific) were used for protein expression

Authentication

Commercial insect cells for protein expression are authenticated based on their general features including morphology and growth condition, as the manufacturer suggested. The cell lines were not authenticated for this research again.

Mycoplasma contamination

All cell lines were not tested for mycoplasma contamination

Commonly misidentified lines
(See ICLAC register)

There is no misidentified lines

## Plants

| | |
|---|---|
| Seed stocks | Plants are not relevant to other experiments |
| Novel plant genotypes | Plants are not relevant to other experiments |
| Authentication | Plants are not relevant to other experiments |
